# Sub-millimeter endoscope demonstrates feasibility of *in vivo* reflectance imaging, fluorescence imaging, and cell collection in the fallopian tubes

**DOI:** 10.1117/1.JBO.26.7.076001

**Published:** 2021-07-02

**Authors:** Ricky Cordova, Kelli Kiekens, Susan Burrell, William Drake, Zaynah Kmeid, Photini Rice, Andrew Rocha, Sebastian Diaz, Shigehiro Yamada, Michael Yozwiak, Omar L. Nelson, Gustavo C. Rodriguez, John Heusinkveld, Ie-Ming Shih, David S. Alberts, Jennifer K. Barton

**Affiliations:** a University of Arizona, Department of Biomedical Engineering, Tucson, Arizona, United States; b Johns Hopkins University, Department of Biomedical Engineering, Baltimore, Maryland, United States; c University of Arizona, Department of Medicine, Tucson, Arizona, United States; d NorthShore University HealthSystem, Evanston, Illinois, United States; e University of Chicago, Pritzker School of Medicine, Chicago, Illinois, United States; f Banner–University Medical Center, Tucson, Arizona, United States

**Keywords:** endoscope, ovarian cancer, fallopian tubes, serous tubal intraepithelial carcinoma, fluorescence, biopsy

## Abstract

**Significance:** Most cases of high-grade serous ovarian carcinoma originate as serous tubal intraepithelial carcinoma (STIC) lesions in the fallopian tube epithelium (FTE), enabling early endoscopic detection.

**Aim:** The cell-acquiring fallopian endoscope (CAFE) was built to meet requirements for locating potentially pathological tissue indicated by an alteration in autofluorescence or presence of a targeted fluorophore. A channel was included for directed scrape biopsy of cells from regions of interest.

**Approach:** Imaging resolution and fluorescence sensitivity were measured using a standard resolution target and fluorescence standards, respectively. A prototype was tested in *ex vivo* tissue, and collected cells were counted and processed.

**Results:** Measured imaging resolution was 88  μm at a 5-mm distance, and full field of view was ∼45  deg in air. Reflectance and fluorescence images in *ex vivo* porcine reproductive tracts were captured, and fit through human tracts was verified. Hemocytometry counts showed that on the order of $10^{5}\text{ cells}$ per scrape biopsy could be collected from *ex vivo* porcine tissue.

**Conclusions:** All requirements for viewing STIC in the FTE were met, and collected cell counts exceeded input requirements for relevant analyses. Our benchtop findings suggest the potential utility of the CAFE device for *in vivo* imaging and cell collection in future clinical trials.

## 1 Introduction

### 1.1 Origin and Characteristics of Early Stage Ovarian Cancer

Due to nonspecific clinical symptoms and a lack of effective screening methods, it is exceedingly difficult to diagnose ovarian carcinoma in its early stages.^1^^,^^2^ Because of the low sensitivity and/or positive predictive value of bimanual ovarian palpation, carbohydrate antigen 125 (CA125) blood tests, and transvaginal ultrasound, even when performed in combination, screening for ovarian cancer can require invasive surgery and removal of the ovaries to make a definitive diagnosis.^3^^–^^7^ As a result, 79% of cases are not discovered until they are already at an advanced stage when five-year survival rates are as low as 29%.^1^^,^^8^^,^^9^ A large number of risk factors for developing epithelial ovarian cancers have been identified, including a family or personal history of breast or ovarian cancer, gene alterations (e.g., BRCA1/2), reproductive history, and advanced age.^8^^,^^10^^–^^13^ Patients at known risk may be counseled to undergo a prophylactic bilateral salpingo-oophorectomy, which reduces ovarian cancer risk by up to 81%, but results in immediate onset symptoms of postmenopause and infertility for premenopausal patients, as well as an increased risk of osteoporosis and cardiovascular mortality long-term.^14^^–^^16^

Mounting evidence suggests that most cases of high-grade serous ovarian carcinoma originate as serous tubal intraepithelial carcinoma (STIC) lesions in the fallopian tube epithelium (FTE), rather than lesions in the ovarian surface epithelium.^17^^–^^20^ STIC lesions, whether invasive or noninvasive, may spread through exfoliation onto the surface of the ovary or into the peritoneal cavity.^21^ Modeling of the growth and progression of these lesions also suggests that there may be a window of detection lasting several years before they metastasize and advance to ovarian cancer.^22^^,^^23^ These findings suggest that a device capable of detecting STIC lesions in the fallopian tubes may enable early stage detection of ovarian cancer. For cancer caught at this stage in the fallopian tube, five-year survival rates may be 95% or greater, and treatment may require neither radical surgery nor adjuvant chemotherapy.^1^^,^^6^^,^^21^

STIC lesions present as clusters of cytologically malignant epithelial cells up to hundreds of microns in diameter that replace normal fallopian tube mucosa, most commonly, but not always, involving the fimbria.^24^^,^^25^ These lesions are smaller than what can typically be seen by whole-body (magnetic resonance imaging, computed tomography, or positron emission tomography) or ultrasound imaging modalities.^7^ This small size of STIC suggests that any visualization method must have a resolution of tens of micron. Additionally, the method should ideally be non-ionizing and comprehensively visualize the entire 11- to 12-cm length of the fallopian tube, as STIC may occur at any location.^7^^,^^22^^,^^24^^–^^26^

Generalizable hallmarks of malignant transformation include the breakdown of collagen and remodeling of tissue, increased cellular metabolism, and increased vascularization.^22^^,^^24^^,^^25^ Therefore, the optical (reflectance and autofluorescence) signature of STIC lesions is expected to be altered.^22^^,^^27^ Changes in the abundance and accessibility of cell surface receptors and extracellular proteins also occur in STIC and subsequent ovarian cancer, including folate receptors and laminin-C1 proteins, which may be targeted with fluorescence-tagged ligands.^28^^,^^29^ STIC lesions are also characterized by unique cellular and molecular features, and thus could be identified with the use of various cytological or omics analyses.^17^^,^^18^^,^^24^^,^^25^ These characteristics indicate that a combination of reflectance imaging, fluorescence imaging, and guided biopsy of epithelial cells from suspect lesions in the fallopian tube may be used to identify STIC before it metastasizes to the ovarian surface epithelium. Such a screening method, designed for the detection of STIC, may also be widely applicable to the detection of other pathology and tubal disease that presents in the FTE or tubal lumen.

### 1.2 Methods for Early Detection of Ovarian Cancer

Any early detection method should be minimally invasive and have high positive predictive value (PPV). The most promising results to date come from serial measurement of CA125 levels and application of the risk of ovarian cancer algorithm (ROCA), followed by transvaginal ultrasound in women at high risk.^5^ Still, the PPV with ROCA is only 10.8% for ovarian or fallopian tube cancers.^30^ Optical imaging is a relatively inexpensive, non-ionizing alternative to whole-body imaging and ultrasound that can detect either endogenous changes in reflectance and autofluorescence or the presence of contrast agents and fluorophores targeted to disease-related biomarkers.^22^^,^^27^^,^^28^^,^^31^^–^^33^ An optical system using reflectance and fluorescence imaging achieved high sensitivity, specificity, and negative predictive value for the detection of clinically occult and cancer-related lesions within *ex vivo* fallopian tube tissue. However, it had high false-positive rates and therefore low PPV.^22^ These results suggest that reflectance and autofluorescence may be best utilized for identification of suspicious areas, to be followed by confirmation of disease with tissue sample acquisition for pathological, cytological, or omics-based analyses.^28^^,^^34^

The major drawback of optical imaging, as with biopsy, is the need to gain physical access to the tissue of interest. Because the uterotubal ostium has a diameter <1  mm across at its narrowest point, size constraints have previously prevented the inclusion of both optical imaging and biopsy in a single endoscopic system capable of operating within the tubal lumen without surgical access.^26^^,^^35^^–^^39^ However, advances in optics and photonics have led to the progressive miniaturization of fibers, lenses, light sources, detectors, and other imaging components, which has resulted in new access to narrow anatomical channels for multifunctional optical endoscopes. Simple optical endoscopes capable of navigating the fallopian tube lumen have been demonstrated *in vivo*, but they have primarily been used for the assessment of tubal patency and identification of stenoses with white-light imaging.^36^^–^^40^ They have had outer diameters as small as 0.50 mm and captured images through a 2000- to 3000-element fiber bundle with optics to obtain up to 50× magnification.^36^^–^^39^ To distend the fallopian tube for improved imaging performance, these devices typically used either a liquid flush, or a linear-everting balloon.^35^^,^^39^ Because these falloposcopes often encountered challenges such as intense light reflections, lenses soiled by floating debris, and insufficient lumen distension, their use in routine clinical practice remains limited.^35^^,^^39^^,^^41^ Still, these simple falloposcopes could sometimes identify tubal causes of infertility.^35^^,^^39^^,^^41^ Recently, a 1.2-mm-diameter scanning-fiber endoscope was utilized for high-resolution white-light imaging of the fallopian tube.^42^ The endoscope had difficulty entering and traversing the length of the tube, possibly due to the relatively large diameter of the endoscope.

Advancement of manufacturing technology, miniaturization of mechanical components, and the development of new, flexible, sterilizable, and biocompatible materials has allowed for the construction of devices such as the Cytuity™ catheter, which is capable of *in vivo* cell collection from the entire proximal portion of the fallopian tube.^43^ However, this device does not include imaging nor enable directed sampling of abnormal tissue. Thus, flexible, steerable biopsy devices, and multimodal optical endoscopes exist that are capable of operation within the fallopian tube lumen, yet no falloposcopic device has included imaging and biopsy together for the comprehensive detection of STIC and other pathology in the FTE.

Here, we present the design, construction, and feasibility testing of a flexible and steerable submillimeter-diameter endoscope capable of multispectral reflectance imaging, multispectral fluorescence imaging, and guided cell collection in the fallopian tubes. We describe the design and construction of our falloposcope based on clinical requirements for detecting STIC, detail the experimental testing of the optical imaging and cell collection subsystems, and summarize the results, which demonstrate that the device is capable of capturing images of adequate resolution for detecting STIC, as well as collecting a sufficient number of cells from the FTE for relevant downstream analyses and diagnosis.

## 2 Methods

### 2.1 Clinical Requirements and System Specifications

Table 1 outlines the clinical requirements and technical specifications for a falloposcope capable of imaging and cell collection. Although these requirements were primarily developed with the goal of detecting STIC, they are also applicable to the detection of other tubal pathology including salpingitis, tubal occlusions, polyps, tumors, and other endotubal lesions or disease.^44^^,^^45^

**Table 1:** Requirements for the design of a minimally invasive, optical endoscope capable of both imaging and cell collection for in vivo detection of STIC within the FTE.

Category	Specification	Requirement	Reason
Mechanical	Outer diameter	≤0.9 mm	Insert through uterotubal ostium diameter as narrow as 1 mm
	Restricted diameter length	≥11 cm	Traverse fallopian tube length of 11 to 12 cm
	Total insertable length	≥70 cm	Traverse entire reproductive tract.
	Flexibility	≤30-mm bend radius	Follow the curvature of the fallopian tube
	Steerability	60 deg	Follow the curvature of the fallopian tube; visualize the fimbria and entire circumference of the tube
	Tube distention	Keep tissue from obstructing optics	Avoid intense light reflections from occlusive tissue and debris
Imaging	Resolution	≤100 μm at working distance of 5 mm	Resolve STIC lesions at typical working distance
	Field of view	>45 deg	Visualize walls of FT
	Fluorescence sensitivity	Detect autofluorescence in ≤0.1 s imaging time	Visualize autofluorescence without undue motion artifact
Illumination	Illumination wavelength	Narrowband blue-green	Identify alterations in natural autofluorescence, enhance hemoglobin contrast in reflection
	Illumination wavelength	Narrowband red	Identify longer-wavelength excited exogenous fluorescence agent
	Illumination wavelength	White-light or RGB	Enable navigation
	Illumination angle	≥45 deg	Illuminate entire field of view
Cell collection	Cellution sample amount	>100 cells	Fulfill sample size requirement for karyometry and most -omics analyses
	Cell type collected	Primarily FTE	Obtain cell type most useful for diagnosis of STIC
Safety	Laser power	<3.2 mW for our geometry	Meet ANSI standard Z136.1
	System electrical leakage current	≤100 μA under normal working conditions	Meet clinical electrical safety requirement IEC 60601-1
	Tissue damage	Biopsy minimally traumatic	Minimize damage to tissue deep to epithelium, avoid perforation
	Sterilizability	No detectable *C. difficile* or *E. cloacae* bacteria after inoculation and sterilization	Meet Laboratory Sciences of Arizona clinical infection prevention standards for sterilization of the single-use device before use with patients/tissue
Clinical considerations	Invasiveness	Office-based or outpatient procedure	Reduce patient morbidity, cost

### 2.2 Operational Procedure

**Fig. 1:**
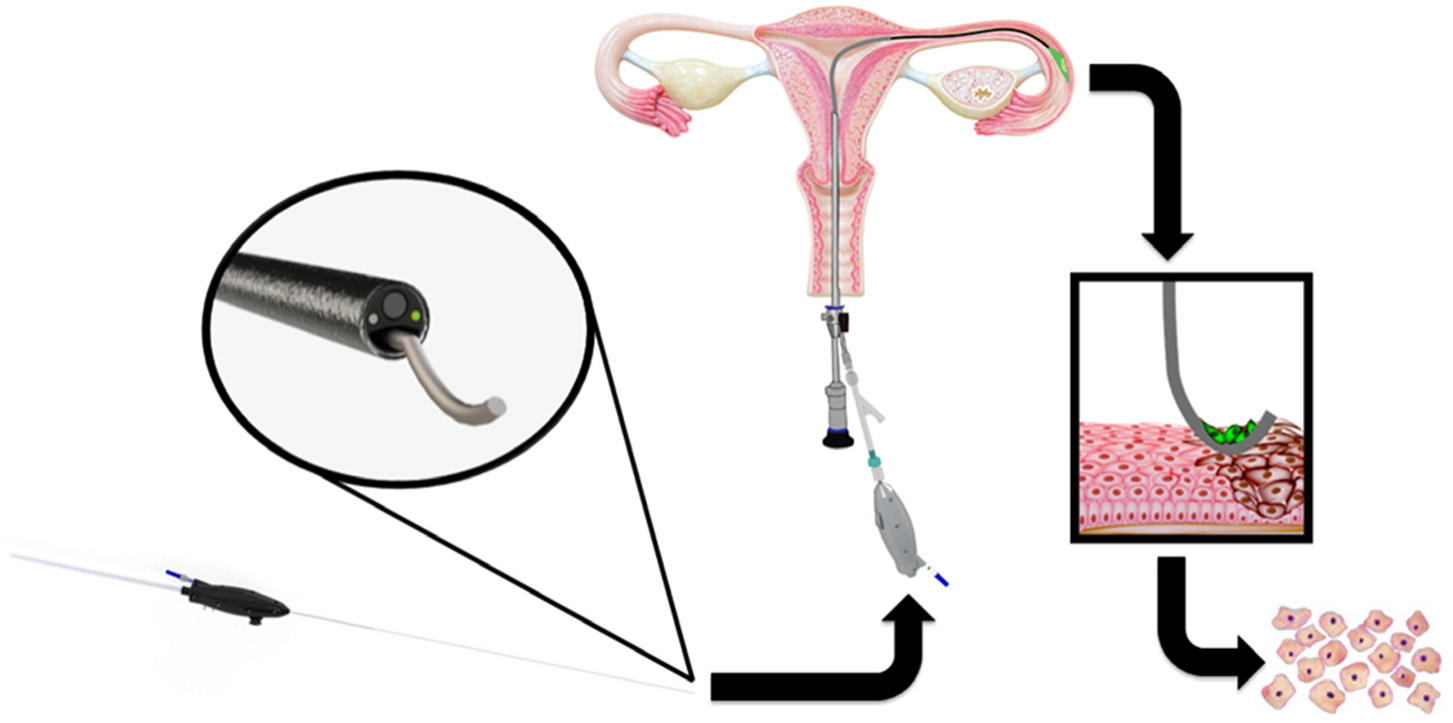
The CAFE is inserted into the uterus and navigated through the fallopian tubes to locate sites of potentially pathological tissue. A scrape biopsy of cells from the region of interest is obtained with the cell collection wire, and the cells can be used for further analysis.

The cell-acquiring fallopian endoscope (CAFE) was designed to meet all of the requirements outlined in Table 1 and be compatible with as much low-cost, standard instrumentation as possible, making it operationally straightforward for use in an outpatient procedure. The endoscope is small enough to be inserted into the uterus through a standard hysteroscope and directed to the uterotubal ostium using a standard salpingography introducing catheter. A hemostasis valve Y-connector placed on the introducing catheter allows for the delivery of liquid or gas as needed to distend the fallopian tube around the CAFE. The CAFE can be navigated through the tubal lumen and used to locate sites of potentially cancerous tissue, as indicated by changes in the optical signature of the tissue. These signs include an alteration in natural tissue reflectance or autofluorescence, or an increase in targeted fluorescence within the FTE.^22^^,^^27^^,^^28^^,^^31^ A curved cell collection wire can be extended from the distal end of the CAFE to obtain a directed scrape biopsy of cells from regions of interest, and then retracted into the endoscope as it is withdrawn from the patient. After the conclusion of the procedure, the cells can be dissociated from the wire to yield material usable for confirming visual predictions with karyometry, proteomics, and other cytological or omics analyses used for the diagnosis of cancer and other pathology.^17^^,^^18^^,^^24^^,^^25^^,^^34^ This procedure is shown in Fig. 1.

### 2.3 Falloposcope System Design

**Fig. 2:**
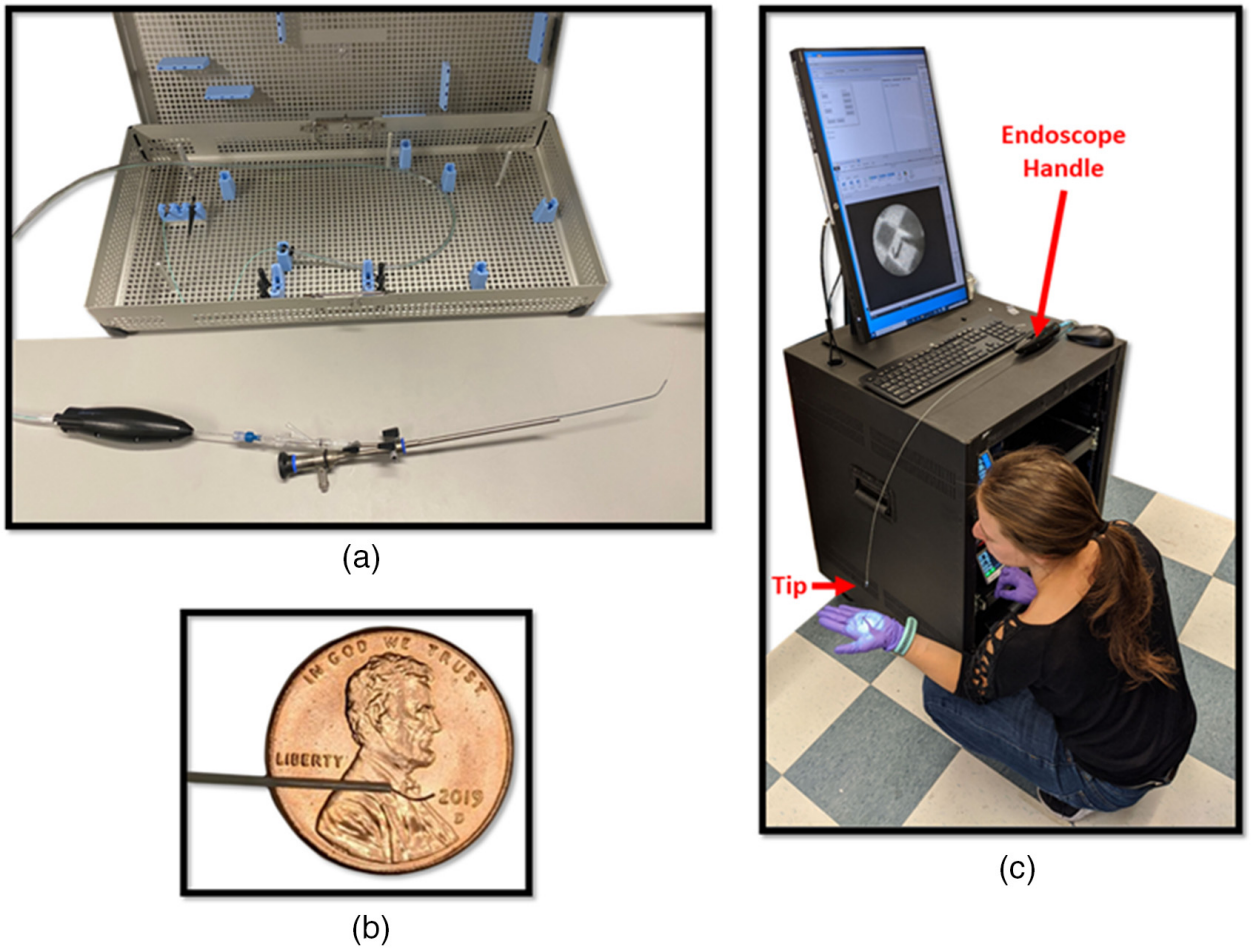
(a) Sterilization case and prototype handheld endoscope inserted into the introducing catheter and hysteroscope. (b) Distal tip of the prototype endoscope with the cell collection wire extended. (c) Full prototype system.

The CAFE falloposcope system consists of a single-use, handheld endoscope that is inserted into the patient, and a reusable, portable console that contains all of the powered components for illumination and imaging. Connection between the console and endoscope occurs at the top surface of the console inside a protective connection box. The CAFE is designed to be inserted through a standard hysteroscope and introducing catheter. Photographs of the completed prototype system are shown in Fig. 2.

#### 2.3.1 Console

**Fig. 3:**
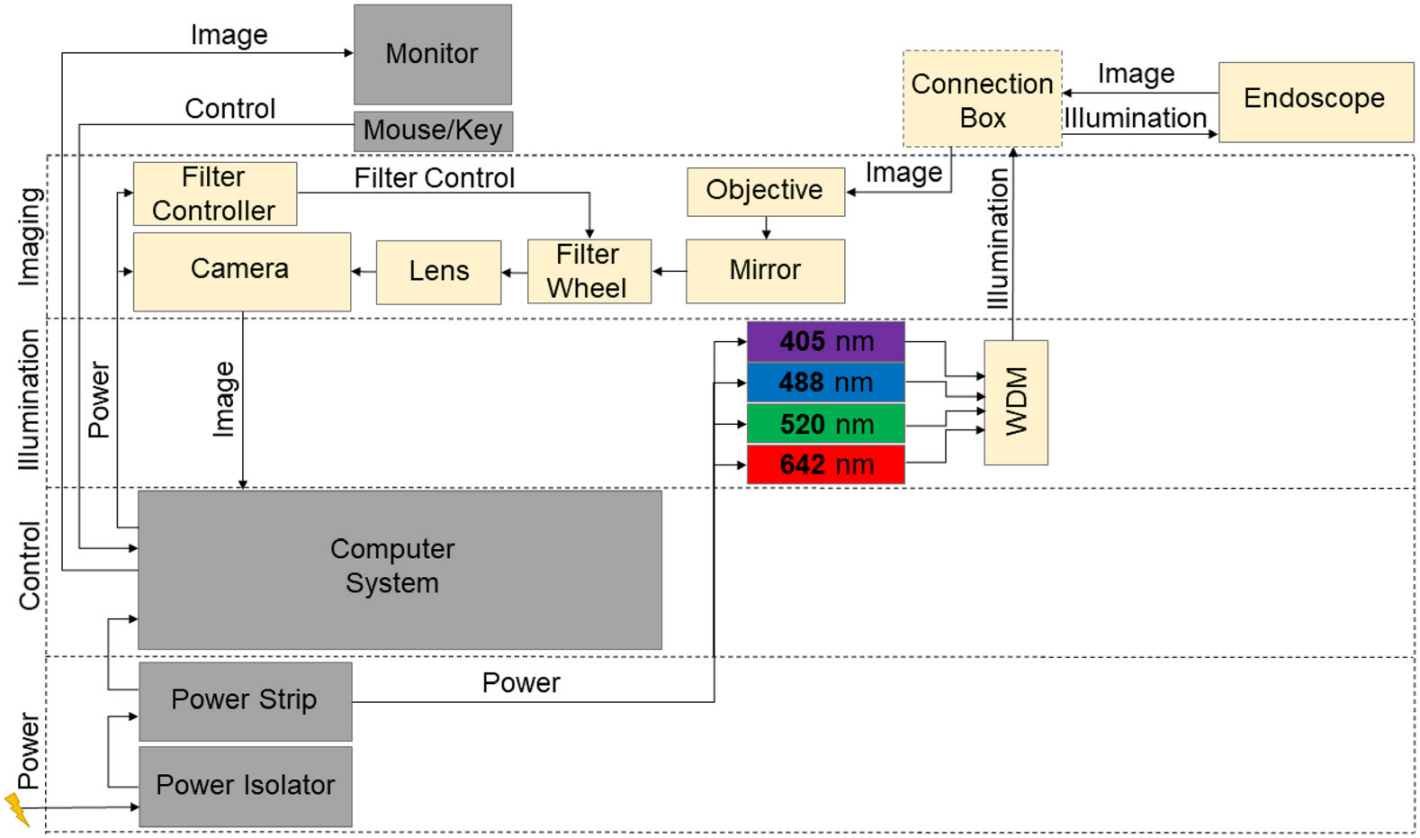
Block diagram illustrating the contents of the CAFE console, responsible for power, control, illumination, and imaging, represented in sections from bottom to top. Components shown in light yellow make up the illumination and imaging subsystems.

The console is a modified, portable equipment rack [Middle Atlantic Products, part number (PN): PTRK-21, Fairfield, New Jersey], containing all power management, computing, illumination, and detection components. A block diagram of these items is shown in Fig. 3. A power isolator [Tripp Lite, PN: IS1000HG 100W Isolation Transformer, Chicago, Illinois] assures conformity with electrical leakage considerations. A computer (HP, Z2 Mini G4 Workstation, Palo Alto, California) with a monitor, keyboard, and mouse enables control of the imaging components, real-time visualization through the endoscope, and storage of captured images and videos. The console houses the illumination sources for imaging, which are four fiber-coupled laser diode sources (Thorlabs, PN: LP642-SF20, LP520-SF15, LP488-SF20, and LP405-SF10, Trenton, New Jersey) operating at emission wavelengths of 405, 488, 520, and 642 nm. These wavelengths are useful for distinguishing the visual features of healthy and diseased tissue with reflectance and fluorescence imaging, and they are readily available as commercial, off-the-shelf components.^22^^,^^27^^,^^28^^,^^31^ The four diodes can be selectively turned on and off for reflectance and fluorescence imaging at each individual wavelength, or multiplexed through a custom, fiber-coupled wavelength division multiplexer [OZ Optics, Ontario, Canada] for imaging with any combination of the four sources. Currently, each laser source is controlled and switched manually using the touchscreen interface of its laser diode driver [Thorlabs, PN: CLD1010LP], but this could be automated in the future. Laser illumination is carried to the endoscope tip by a single illumination fiber. Narrowband reflectance imaging can be performed to increase the contrast of all vasculature in the tissue, whereas fluorescence imaging can be used to detect either targeted fluorophores or metabolic and microstructural changes in the tissue.^22^ Pseudo-white-light imaging can be accomplished by overlaying reflectance images taken sequentially at each wavelength. The total laser power incident upon the tissue must comply with ANSI standard Z136.1. For continuous illumination with visible wavelengths, radiant exposure is limited to $0.2\;W/{\mathrm{cm}}^{2}$, corresponding to about 3.2-mW total laser power emitted from the tip of the endoscope at a working distance of 5 mm from the tissue.

The image captured by the handheld endoscope is relayed to the console through a fiber bundle. The end of the fiber bundle is imaged by a 40× plan achromatic microscope objective [Olympus, PN: PLN-40×, Center Valley, Pennsylvania] with a 0.65 NA, and the image is passed through a fold mirror, filter wheel [Thorlabs, PN: ELL9K], and an achromatic lens [Melles Griot, PN: 01LA0119, Rochester, New York] to focus on a six-megapixel monochrome CCD camera [Retiga, PN: R6, Surrey, BC, Canada] with 4.54  μm pixels, thermoelectric cooling, and a 75% peak quantum efficiency. The filter wheel positions an appropriate long-pass filter in front of the camera for fluorescence imaging with each laser diode. This includes a 450-nm long-pass filter [Thorlabs, PN: FEL0450] for the 405-nm laser source, a 500-nm long-pass filter [Thorlabs, PN: FEL0500] for the 488-nm laser source, a 550-nm long-pass filter [Thorlabs, PN: FEL0550] for the 520-nm laser source, and a 650-nm long-pass filter [Thorlabs FEL0650] for the 642-nm laser source. One slot in the filter wheel is left empty for reflectance imaging. Ocular software [Teledyne Photometrics, Ocular Image Acquisition Software, Tucson, Arizona] is used to set exposure time, adjust image settings (brightness, contrast, etc.), capture and save still images, record and save video, add false color, and overlay images for pseudo-white-light image capture and navigation through the fallopian tube. Image acquisition speeds of ∼10 frames per second can be achieved with a 0.100-s exposure time. Separate software [Thorlabs, Elliptec Systems Software] controls the filter wheel and maintains a 450- to 500-ms switching time.

#### 2.3.2 Endoscope

**Fig. 4:**
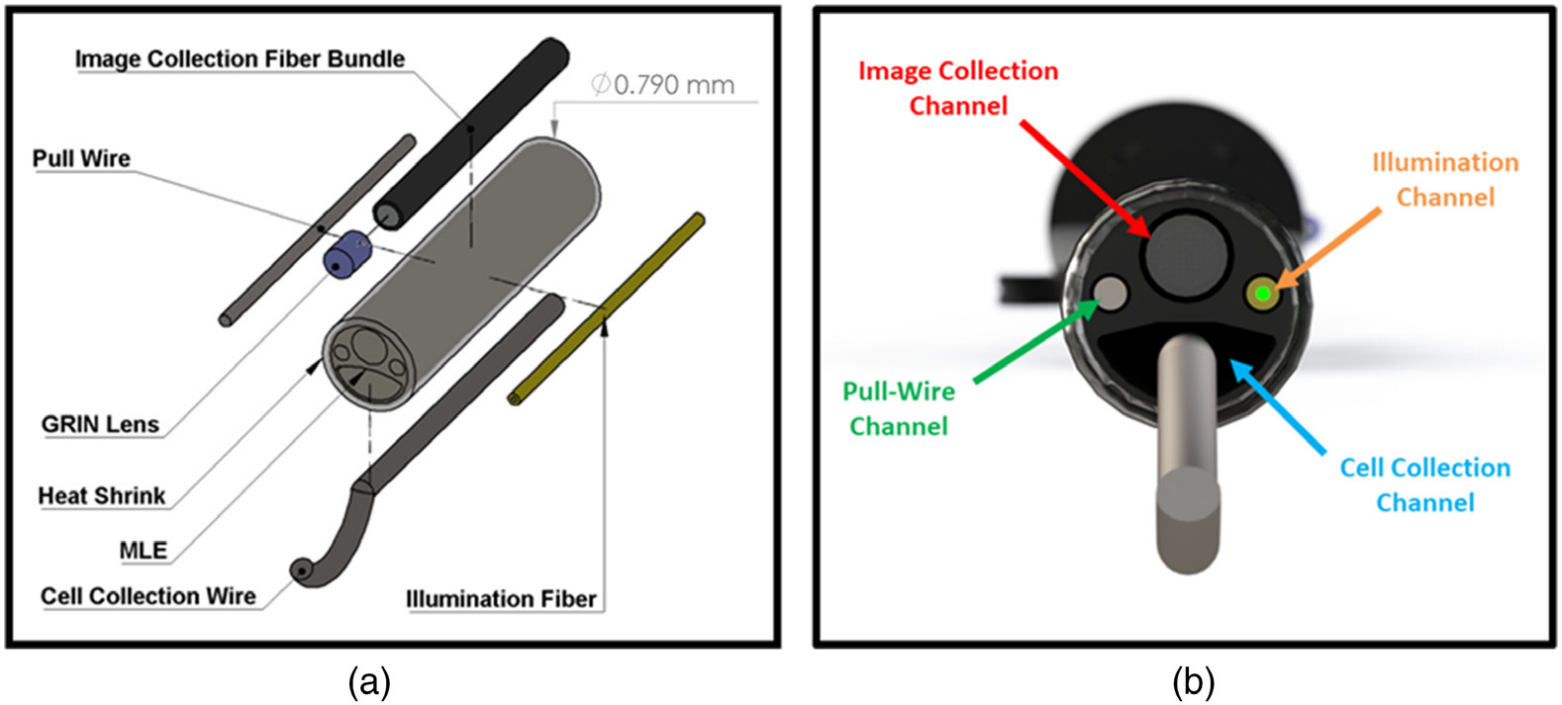
(a) Exploded view of all handheld endoscope components contained within the MLE at its distal tip. (b) Rendered image of the distal tip of the endoscope with the cell collection wire extended and the MLE channels labeled.

The handheld endoscope utilizes many of the same design principles as the single-use, multimodal falloposcope described in a previous publication from our research group.^40^ However, the endoscope previously described did not have cell collection capability. The CAFE has multiple components packed into a 70-cm-long insertable body that is 0.79 mm in diameter and is connected to a handle with unidirectional steering control. The main body is a flexible, 0.71-mm-diameter multi-lumen extrusion (MLE) of polyetheretherketone (PEEK) plastic, which contains four channels of different sizes, shown and labeled in Fig. 4. The image collection channel of the MLE is a circular, 0.30-mm-diameter channel containing a 3000-element image fiber bundle [Fujikura, PN: FIGH-03-200S, Tokyo, Japan] with an attached gradient index (GRIN) lens [GRINtech, PN: GT-IFRL-025-005-50-CC, Jena, Germany]. On each side of the MLE, there are circular channels 0.13 mm in diameter: the pull-wire channel houses a 0.10-mm-diameter, 304 V stainless alloy pull-wire [Fort Wayne Metals, Fort Wayne, IN], whereas the illumination channel houses a 0.10-mm-diameter, teflon-clad, multimode illumination fiber with an 85  μm core diameter and a 0.66 NA [Polymicro Technologies, PN: FSU085100, Phoenix, Arizona]. The pull-wire enables up to 60-deg unidirectional steering at up to a 25-mm bend radius (bend limit of the image collection fiber bundle).

The cell collection channel is 0.26-by-0.57 mm in diameter and has a gibbous shape for housing the curved cell collection wire. This channel, which occupies all remaining space in the endoscope, is too small to accommodate commercially available biopsy devices, baskets, or cytology brushes. A small diameter nitinol wire can be shape-memory formed such that can traverse the small diameter channel, and then assume a gentle curve when extended out the distal end of the endoscope. Various diameters and shapes of wire can be used.

The rest of the handheld endoscope design closely follows the design of the multimodal falloposcope for clinical use detailed in our research group’s previous publication.^40^ In summary, multiple sections of optically transparent, thin-walled, medical grade heat shrink tubing [Nordson Medical Corp., PN: 103-0139, Westlake, Ohio] protect the distal tip of the endoscope, bringing the total outer diameter of the insertable portion of the endoscope to 0.79 mm. Multiple layers of thicker heat shrink [Nordson Medical Corp., PN: 103-0454] tubing are used on the rest of the PEEK MLE to create a biocompatible, smooth, sterilizable surface with an overall diameter of 0.79 mm. A 15.25-cm-long, 1.87-mm-diameter stainless steel hypotube [Vita Needle, Needham, Massachusetts] supports the MLE where it attaches to the endoscope handle.

**Fig. 5:**
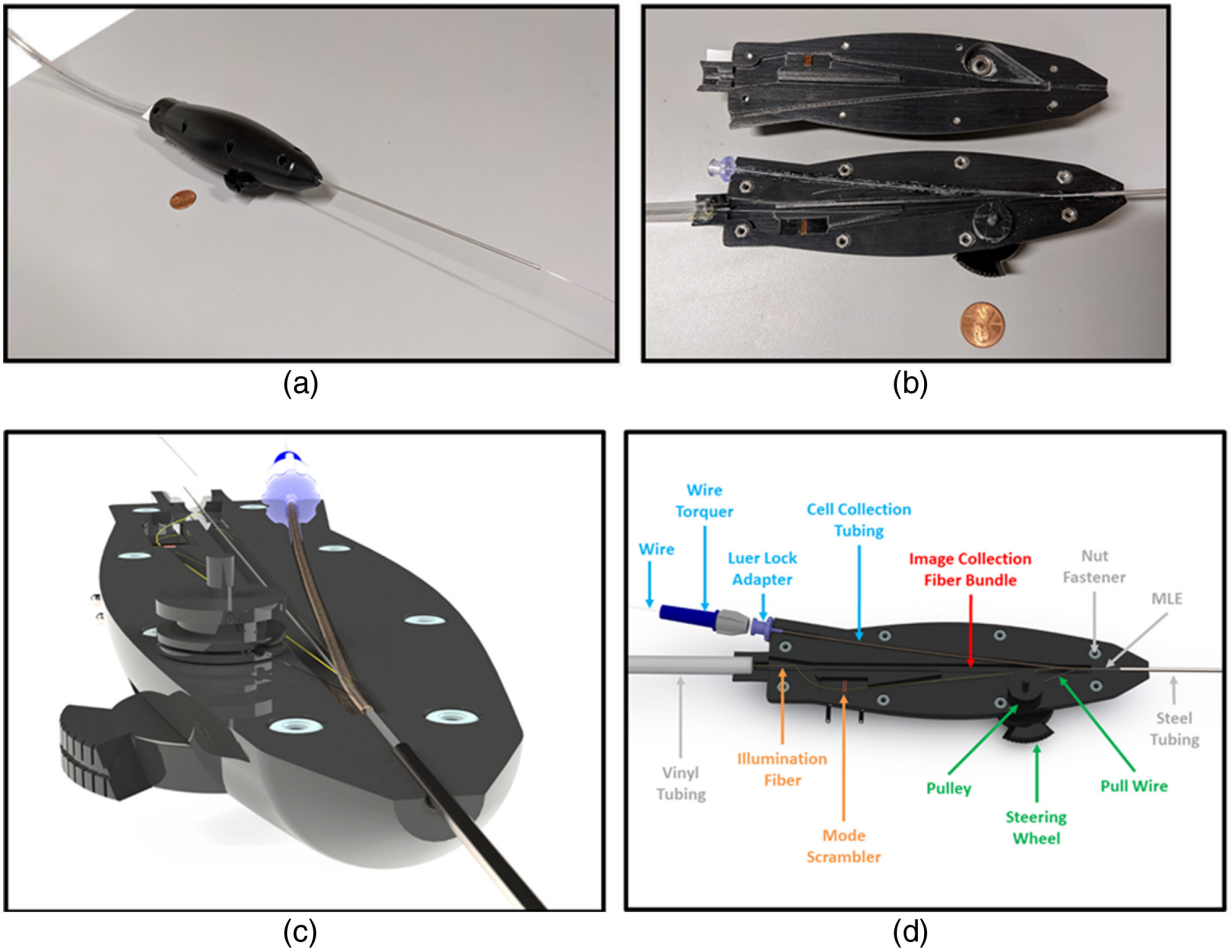
(a) Image of the prototype handheld endoscope. (b) Image of the opened endoscope handle showing all internal components. (c) Rendered image of the components inside of one half of the handheld endoscope. (d) Rendered image of the handheld endoscope with all internal components labeled.

All endoscope components run through an 18-cm-long, 3D-printed, ergonomic handle. Renderings of the handle with all internal components labeled are shown in Fig. 5. The stainless steel pull-wire wraps around a pulley that is attached to a steering wheel on the outside of the handle, enabling unilateral tip deflection. The entire endoscope can be rotated to control the direction of deflection. The cell collection wire runs through a separate channel in the handle that is lined with lubricious tubing and mated to the MLE cell collection channel. A female Luer Lock adapter at the surface of the handle can be used for the delivery of liquid through the cell collection channel when the cell collection wire is not inserted, enabling the clearing of debris from the endoscope optics and dispensing of exogenous fluorophores into the fallopian tube. Maneuverability and control of the cell collection wire by the operator is improved by a wire torquer attachment attached near the proximal end of the wire. The image collection fiber bundle passes through a straight, central channel in the handle, whereas the illumination fiber follows a separate, lateral channel that contains a custom mode scrambler for adjusting the output angle of illumination from the endoscope.^40^ A 3-m long, vinyl-encased cable connects the endoscope to the console.

### 2.4 Experimental Setup and Characterization

A prototype CAFE console and endoscope were constructed and tested to assure they met the requirements for imaging, illumination, and cell collection outlined in Table 1.

#### 2.4.1 Imaging and illumination testing

**Fig. 6:**
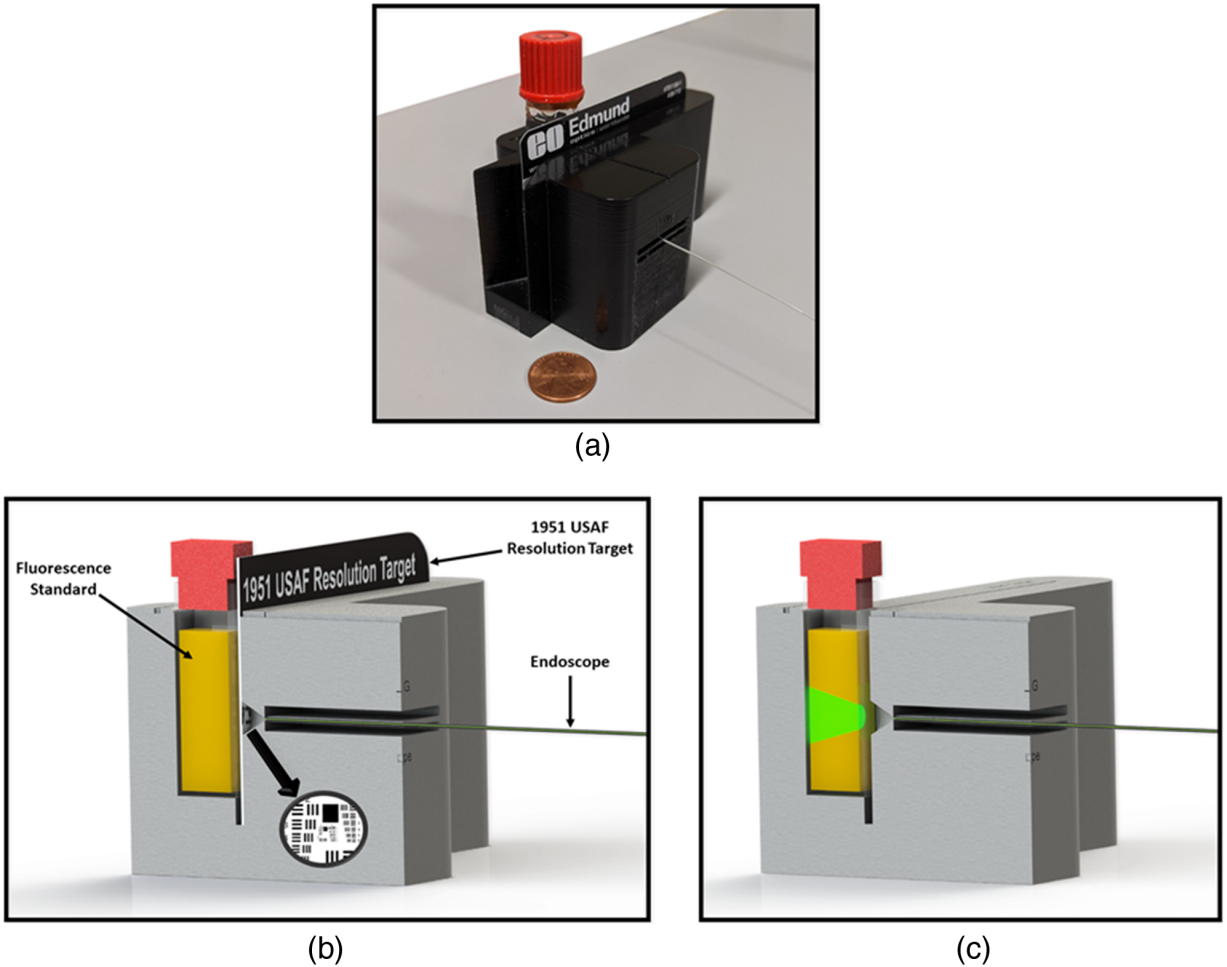
(a) Rendered image of the illumination and imaging test setup with a 1951 USAF Resolution Target and cuvette of liquid fluorescence standard placed in the 3D-printed jig. (b) Cross-section of the test setup with endoscope inserted and components labeled.

As shown in Fig. 6, a 3D-printed, fixed-geometry jig was used for repeatable testing of imaging and illumination in a dark environment. The jig holds the endoscope tip 5 mm from a resolution standard, and 6.75 mm from a 1-cm square cuvette containing a fluorophore. For resolution measurements, the 488-, 520-, and 642-nm laser diodes were turned on simultaneously and images of a standard 1951 USAF Resolution Target were captured. The images were analyzed in ImageJ [NIH, Version 1.8.0_112], defining resolvable elements as those with a minimum modulation contrast of 15% for both horizontal and vertical bars, based on the Rayleigh criterion.^46^ Field of view was measured by imaging a one-line-pair-per-millimeter (lp/mm) target. In ImageJ, the width of one line pair was used to set the scale, and then the distance over the entire imaging circle was measured.

Illumination and fluorescence imaging sensitivity were tested using cuvettes of fluorescent dyes—acridine orange (18-μM dissolved in ethanol), fluorescein (2-μM dissolved in 0.1 M sodium hydroxide), and rhodamine (0.8-μM dissolved in ethanol) – which produce remitted fluorescence signal similar to what is expected from tissue at excitation wavelengths of 405, 488, and 520 nm, respectively. An image was captured with a 100-ms exposure time through the appropriate fluorescence filter. Reflectance and fluorescence images were obtained with and without laser illumination to measure illumination uniformity and signal-to-background ratio. Qualitative analysis of image quality was assessed from reflectance (monochrome, narrow band, and pseudo-white-light) and fluorescence images that were acquired in fresh *ex vivo* porcine fallopian tube tissue. Reflectance and fluorescence images were also captured in *ex vivo* human fallopian tube tissue, but these image acquisition tests were used to assess the feasibility of fitting the endoscope into human fallopian tubes for imaging, rather than generalizable formal analysis of image quality. The human tissue was unsuited for image analysis because all samples acquired as surgical discard from procedures involving salpingectomy were limited to <1  cm in length through our Institutional Review Board human subjects protocol with the Banner University Medical Center Department of Pathology.

#### 2.4.2 Cell collection testing

After measuring illumination and image quality, the cell collection subsystem was tested for its ability to acquire an adequate number of cells from the FTE for downstream cytological and -omics analyses.

Candidate wire designs were constructed and evaluated based on their ability to maximize the number of cells collected from a single tissue scrape in fresh *ex vivo* porcine fallopian tube tissue. Nitinol cell collection wires with all combinations of diameters (178, 203, 223, and 254  μm), finishes (light oxide smooth and etched and polished rough), and distal shapes (quarter-circle and shepherd’s hook) were fabricated. All *ex vivo* tissue was obtained from animals processed at the University of Arizona Food Products and Safety Laboratory immediately after slaughter, and experiments were conducted at room temperature. Each wire was used to scrape the FTE by pressing the endoscope against the fallopian tube wall and rotating the cell collection wire while drawing it back along a 1-cm section of fresh *ex vivo* porcine fallopian tube. Each wire design was tested in sections of isthmus, ampulla, infundibulum, and fimbria tissue. The endoscope was retracted, and the distal tip of the wire was snipped off into a 1.5-ml tube filled with phosphate-buffered saline (PBS). These tubes were centrifuged to remove biopsy material and create a cell pellet. The number of cells collected was counted using a hemocytometer. Using both unpaired student T-tests and one-way analysis of variance tests, we performed statistical analyses to test for a statistically significant (P<0.05) difference in the number of cells collected by each wire design.

Scraped fallopian tubes were fixed in 10% neutral buffered formalin, embedded in paraffin wax, sectioned, stained with hematoxylin and eosin (H&E), and imaged to investigate whether the cell collection procedure had appropriately removed the epithelial layer from within the tubal lumen without causing damage to deeper tissues.

To further optimize the collected biopsy material for karyometry and other cytological analyses that require a monolayer of individual cells, rather than tissue pieces or clumps of cells, an alternative processing method was developed. Wires were snipped into tubes containing a solution of collagenase type I enzymes (Thermo Fisher Scientific, PN: 17018029, Waltham, Massachusetts) in Hank’s Balanced Salt Solution (HBSS) and left to incubate at 37°C for 1.5 hours. The tubes were centrifuged for 10 min at 300 G to remove any material remaining on the wire. The wire piece was removed from each tube, and the biopsied material was resuspended in HBSS. To verify that we collected both ciliated cells and secretory cells (the cells most closely associated with the development of STIC lesions and ovarian cancer), a portion of collected samples from fresh, *ex vivo* ovine tissue was stained using standard immunofluorescence protocols. DAPI nuclear marker was used to stain the total cell population. Secretory cells were identified with anti-PAX8 antibodies [Cell Signaling Technology, PN: 59019S, Danvers, Massachusetts], and ciliated cells were identified with anti-acetylated-tubulin antibodies (primary cilia and multi-cilia marker) (Millipore Sigma, PN: T7451, St. Louis, Missouri). Dissociated FTE cells were also stained with anti-vimentin antibodies (Abcam, PN: ab92547, Cambridge) and anti-cytokeratin-7 antibodies (Abcam, PN: ab9377). The remaining samples were fixed with 10% neutral buffered formalin, stained with H&E, and imaged using a high-resolution microscope to verify that the biopsy material retrieved by the CAFE and dissociated with this procedure produced individual cells of sufficient quantity and quality for karyometric analysis.

## 3 Results

### 3.1 Imaging and Illumination

**Fig. 7:**
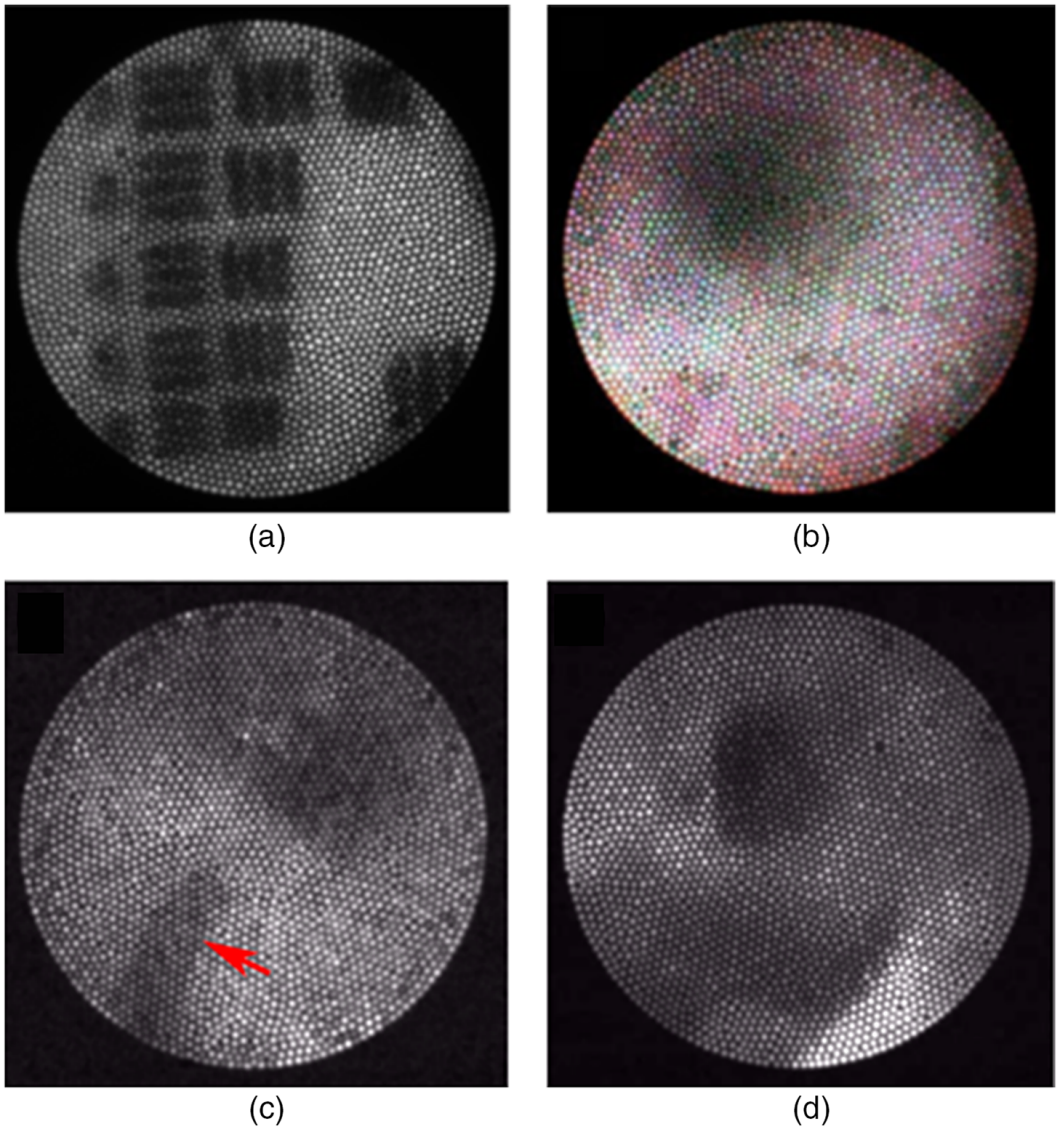
(a) Pseudo-white-light image of Group 2 of the 1951 USAF Resolution Target. (b) Pseudo-white-light, false-colored image captured during navigation in an *ex vivo* porcine fallopian tube. (c) Monochrome reflectance image showing the cell collection wire (indicated by red arrow) extended during scrape biopsy in an *ex vivo* porcine fallopian tube. (d) Monochrome fluorescence image taken with 488-nm illumination and a 500-nm long-pass filter in an *ex vivo* porcine fallopian tube (Video 1, MP4, 14 MB [URL: https://doi.org/10.1117/1.JBO.26.7.076001.1).

All imaging requirements in Table 1 were met. Analysis of the images of the 1951 USAF Resolution Target [as shown in Fig. 7(a)] showed that the smallest resolvable element was group 2 element 4 when acquired from a distance of 5 mm from the target. This corresponds to a resolution of 88.4  μm. The full field of view was ∼45  deg in air. Fluorescence emission across the field of view varied with about a ratio of seven between the brightest patch and darkest patch of the image, but in all cases the signal-to-background ratio was at least 2. Each patch was defined as seven pixels from the optical fiber bundle.

Feasibility testing in *ex vivo* human fallopian tube tissue confirmed that the endoscope successfully fit through sections of the isthmus and ampulla for imaging. Reflectance and fluorescence images were acquired in *ex vivo* porcine fallopian tube tissue as shown in Fig. 7. This includes pseudo-white-light images captured within the tubal lumen during navigation [shown in Fig. 7(b)], monochrome reflectance images captured during cell collection [shown in Fig. 7(c)], and fluorescence images captured during navigation [shown in Fig. 7(d)]. These images were processed with a 1 pixel-width Gaussian blur function to de-emphasize the fiber bundle honeycomb appearance. Figure 7(d) is a frame from a video captured in fluorescence imaging tests, which demonstrates translation of the endoscope during navigation and visualization of fluorescent dye (Video 7D). We encountered some problems with intense light reflections and floating debris previously reported in other falloposcopy procedures.^35^^,^^39^^,^^42^ However, these phenomena were managed with the introduction of air and saline into the introducing catheter and cell collection tubing, respectively, for distending the tube and clearing debris from the optics while imaging.

### 3.2 Cell Collection

Statistical analysis of the number of cells collected with Nitinol wires of various designs revealed that there was no statistically significant difference in the average number of cells collected by wires of each tested shape (P=0.92, Table S1 in the Supplementary Material) diameter (P=0.84, Table S2 in the Supplementary Material), and surface finish (P=0.87, Table S3 in the Supplementary Material), nor in the number of cells collected from isthmus, ampulla, or infundibulum sections of *ex vivo* porcine fallopian tubes. In all cases, on the order of $10^{5}\text{ cells}$ per tissue scrape could be obtained.

**Fig. 8:**
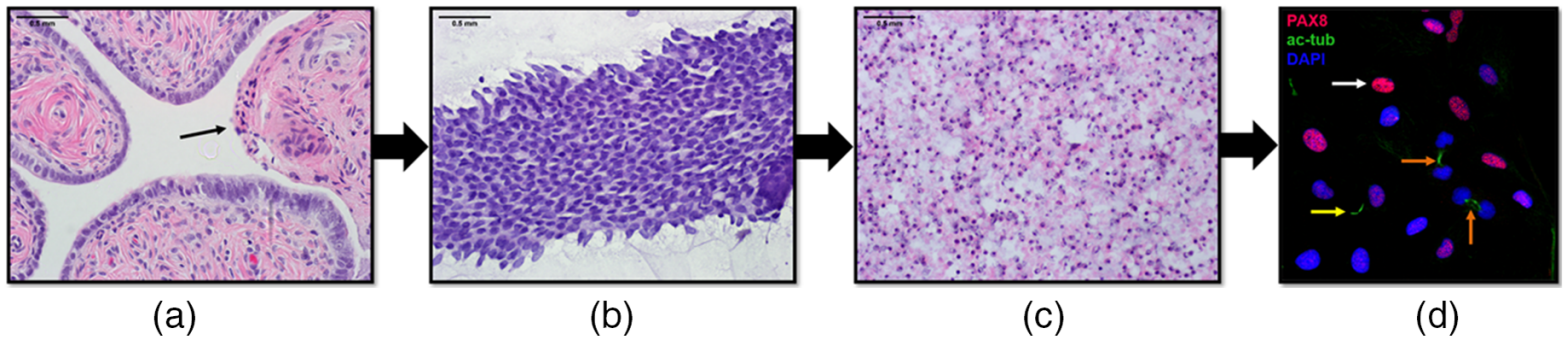
(a) H&E-stained cross-section of biopsied fallopian tube tissue showing the atraumatic removal of the epithelial layer of cells on the right side of the image. (b) H&E-stained clump of tissue prior to the dissociation procedure. (c) H&E-stained cells spread in monolayer on a slide after dissociation. (d) Image from immunofluorescence experiments on dissociated cells showing secretory cells with labeled PAX8 (magenta), ciliated cells labeled with acetylated tubulin (green), and all nuclei labeled with DAPI (blue). Arrows are used to indicate secretory cells (white arrow), primary cilia (yellow arrow) on secretory cells, and multi-cilia (orange arrow) on ciliated cells collected from the FTE.

Figure 8 shows images of samples from the test biopsy procedures. As shown in Fig. 8(a), H&E-stained cross-sections of scraped fallopian tube show clean removal of the FTE with no histologically apparent damage to deeper tissues. Tissue clumps collected prior to dissociation and individual cells retrieved after dissociation are shown in Figs. 8(b) and 8(c), respectively. Figure 8(d) shows dissociated secretory and ciliated epithelial cells with nuclear material, PAX8, and acetylated tubulin labeled using standard immunofluorescence procedures. In addition, Fig. S1 in the Supplementary Material shows dissociated FTE cells with stained vimentin and cytokeratin-7, confirming that they are of epithelial origin. Multiple samples suggest that we are collecting mostly intact epithelial cells from the FTE in each biopsy procedure, sufficient in quantity and quality for multiple types of analyses.

## 4 Discussion

All requirements set forth in Table 1 were met by our prototype endoscope system. Measured resolution and test images captured with the CAFE suggest that the endoscope has sufficient image resolution, illumination spot size, and fluorescence emission sensitivity to potentially enable the detection of STIC lesions and other pathology in the fallopian tube with reflectance and fluorescence imaging. This version the CAFE system currently requires any identification of suspect lesions to be performed by the operating physician, who must use visual feedback from the endoscope to discriminate between normal and abnormal tissue. In the future, however, we hope to include computer vision and machine learning frameworks in custom software for highlighting suspect regions of tissue that show alterations in reflectance and fluorescence intensity outside of the normal or expected range. Testing the CAFE system in *ex vivo* tissue showed the ability to navigate the fallopian tube with manageable, periodic occurrence of intense light reflections, and debris. Background autofluorescence from all parts of the system, including that which may arise from the image fiber bundle and PEEK MLE with 405-nm excitation, was characterized and found to have no significant impact on fluorescence imaging performance with measures of signal-to-background ratio, as described in our results.

In fresh, normal, *ex vivo* fallopian tube tissues, we also showed that it is possible to collect a sufficient number of intact FTE cells for use in morphological, cytological, and omics analyses with a single scrape biopsy from the CAFE device. The current design of the CAFE also allows for the user to either collect an aggregated sample of tissue with a single wire used to scrape multiple sites, or multiple separate samples collected by inserting a new wire before each sampling event. Aggregated samples cannot be separated by location of collection, but this may not be necessary because the diagnosis of STIC or cancer in any portion of the fallopian tube might prompt salpingectomy of that entire tube. Of note, we have yet to establish a precise method of connecting collected samples and images to physical location within the fallopian tube for follow-up screening, but an approximate distance from either end of the tube can be estimated by the distance of the handle from its forward limit.

We will use a wire with diameter of 178  μm (0.007”) and a light oxide surface finish [Component Supply, PN: NW-0070, Sparta, Tennessee] for cell collection since no statistically significant difference was shown in the number of cells collected by wires with diameters of 178  μm (0.007”), 203  μm (0.008”), 223  μm (0.009”), and 254  μm (0.010”), and it is the smallest commercially available diameter that maintains satisfactory pushability and torquability during scraping procedures with the CAFE. Leaving as much room as possible between the outer surface of the wire and the wall of the cell collection channel of the MLE should minimize the number of cells scraped off of the cell collection wire during retraction back into the MLE. We will use the gentle, quarter-circle curve shape, as it is both compact and easy to retract into the cell collection channel of the MLE. The collected cells can be used for a variety of diagnostic tests: they can be smeared on a microscope slide, fixed and stained, pelleted for molecular analysis, or dissociated, filtered, and placed in a monolayer as required for karyometric analysis.

H&E staining of *ex vivo* tissue sections demonstrates that this method of biopsy results in minimal damage to the fallopian tube. As documented in previous clinical studies of falloposcopy procedures, complete perforation or partial dissection of the fallopian tube has not been followed by any sequelae or any need for further intervention.^35^ This observation yields reasonable promise for confirming the safety of the CAFE device in future clinical trials, even if unexpected perforation with the cell collection wire is noted in the future. In summation, our results suggest that the detection of STIC with the CAFE device could be possible in future clinical trials, which could contribute to the development of new, minimally invasive screening methods for the detection of early stage ovarian cancer. If these STIC lesions can be detected in the window of time before they metastasize to the ovary or pelvic cavity, intervention can occur when five-year survival rates are as high as 95% or greater and treatment may require neither radical surgery nor adjuvant chemotherapy.^1^^,^^6^^,^^21^

The cost of manufacturing each of our single-use, handheld endoscopes was kept as low as possible, resulting in a total parts cost of $796 per endoscope. Major contributors to this cost are the 3000-element image fiber bundle and the custom PEEK MLE. We put considerable effort toward minimizing costs because the development of such low-cost, minimally invasive, and effective screening methods is important not only for the reduction of mortality due to ovarian cancer, but also for increasing access to regular health screening that can reduce morbidity in vulnerable populations less likely to seek treatment for gynecological health concerns. With these patient populations in mind, we are continuing to make frugal improvements to the design of the CAFE device as we look to move forward to clinical trials and *in vivo* testing for safety and efficacy in the near future. We are currently planning improvements to this design that utilize higher-resolution optics and lower-profile cell collection wires. This includes versions of the CAFE with either a 10,000-element fiber bundle (0.450-mm diameter) or up to a 160-kilopixel chip-on-tip image sensor ($0.650\times 0.650\times 1.198\;{\mathrm{mm}}^{3}$ package). Custom software designed to utilize frame fusion and image reconstruction methods could also be used to increase the resolution of images captured with either our current CAFE system or an updated chip-on-tip endoscope.^47^ We are also designing updated versions of the CAFE that utilize alternative wire designs for preventing potential perforation of the tube and more easily retracting the wire into the endoscope tip. So far, 14 units of the handheld CAFE endoscope and two units of the reusable console have been completed for testing on additional *ex vivo* human fallopian tube tissue with and without targeted fluorophores or contrast agents for labeling STIC. These tests will be used to inform further changes made to the device before planning pilot feasibility and safety studies in normal, cancer-free patients already undergoing procedures that include cervical dilation with salpingectomy. In consenting patients participating in the study, additional time could be added to the procedure in which the physician would place the CAFE device and introducing catheter through the hysteroscope already inserted in the patient to image and collect cells from the FTE. Because the fallopian tubes from these patients will be removed at the conclusion of the scheduled procedure, this presents a low-risk opportunity to confirm the utility of the CAFE device for *in vivo* imaging and cell collection representative of its potential use in future applications for ovarian cancer screening and more. Such a screening method, designed for the detection of STIC, may also be widely applicable to the detection of other pathology and tubal disease that presents in the FTE or tubal lumen.
