# Supplementary material for: Sub-millimeter endoscope demonstrates feasibility of in vivo reflectance imaging, fluorescence imaging, and cell collection in the fallopian tubes
Source: J Biomed Opt. 2021 Jul 2;26(7):076001. doi: 10.1117/1.JBO.26.7.076001 (PMC8253554; doi:10.1117/1.JBO.26.7.076001)
Supplement: Supplementary file 1 [file JBO_026_076001_SD001.pdf]

Supplementary Material

Cell Collection Wire Comparison Results

Table S1: Hemocytometry counts of cells collected by 0.223-millimeter-diameter, light oxide wires of each shape with a single scrape biopsy. Five separate trials were conducted within fresh *ex vivo* sections of porcine fallopian tube tissue from the ampulla and infundibulum. Average collected cell counts are presented as the mean of the data from all five trials with each wire.

	**Wire Shape**	
	**Gentle Curve**	**J-hook**
FT 1	3.40e+5	1.40e+5
FT 2	2.20e+5	4.40e+5
FT 3	2.40e+5	6.60e+5
FT 4	3.10e+6	1.32e+6
FT 5	1.66e+6	2.64e+6
Average	1.11e+6	1.04e+6

Table S2: Hemocytometry counts of cells collected by light oxide wires of each diameter with a single scrape biopsy. Five separate trials were conducted within fresh *ex vivo* sections of porcine fallopian tube tissue from the ampulla and infundibulum. Average collected cell counts are presented as the mean of the data from all five trials with each wire.

	**Wire Diameter**			
	**178 µm (.007")**	**203 µm (.008")**	**223 µm (.009")**	**254 µm (.010")**
FT 1	4.02e+6	4.77e+6	3.27e+5	1.00e+6
FT 2	1.67e+5	2.00e+4	4.00e+4	4.00e+4
FT 3	6.98e+5	4.20e+6	4.15e+6	5.23e+5
FT 4	9.97e+5	5.27e+6	1.67e+6	9.40e+6
FT 5	7.10e+5	2.08e+6	1.00e+4	6.70e+5
Average	1.32e+06	3.27e+06	1.24e+06	2.33+06

Table S3: Hemocytometry counts of cells collected by 0.178-millimeter-diameter wires of each surface finish with a single scrape biopsy. Ten separate trials were conducted within fresh *ex vivo* sections of porcine fallopian tube tissue from the ampulla and infundibulum. Average collected cell counts are presented as the mean of the data from all ten trials with each wire.

	Surface Finish	
	**Light Oxide**	**Etched & Polished**
FT 1	1.10e+5	1.15e+6
FT 2	1.60e+5	2.52e+6
FT 3	5.90e+5	1.00e+4
FT 4	5.70e+5	3.50e+5
FT 5	6.90e+5	5.40e+5
FT 6	3.30e+5	1.55e+5
FT 7	1.90e+5	2.00e+4
FT 8	3.85e+5	6.00e+4
FT 9	7.00e+5	9.00e+4
FT 10	1.38e+6	4.00e+4
Average	4.47e+5	4.94e+5

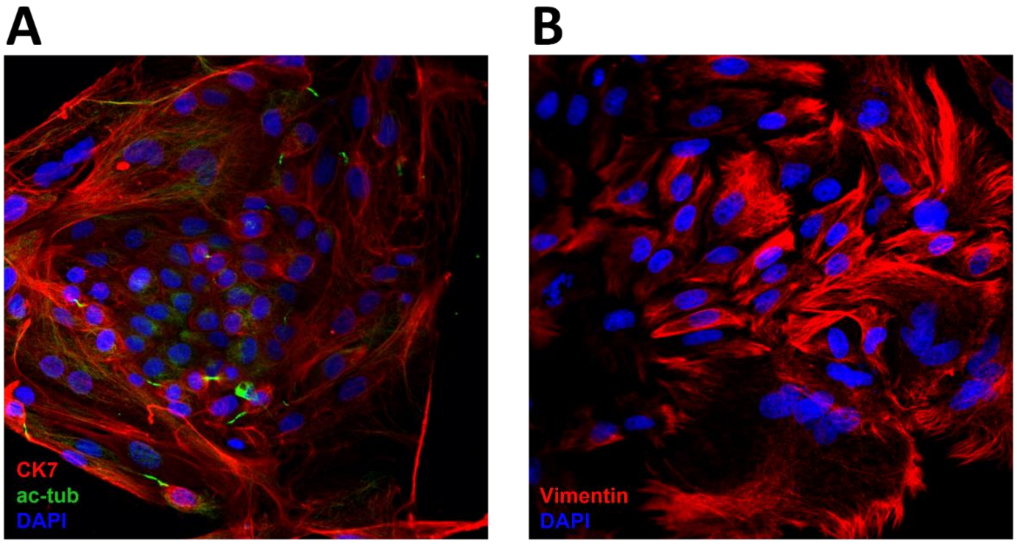

Figure S1: (A) Immunofluorescence image showing dissociated cells with labeled cytokeratin-7 (CK7, red), acetylated tubulin (ac-tub, green), and nuclear material (DAPI, blue). (B) Immunofluorescence image showing dissociated FTE cells with labeled vimentin (red) and nuclear material (DAPI, blue).
